# Novel Models for Accurate Estimation of Air–Blood Partitioning: Applications to Individual Compounds and Complex Mixtures of Neutral Organic Compounds

**DOI:** 10.1021/acs.jcim.3c01288

**Published:** 2023-11-13

**Authors:** Ahmad Aakash, Ramsha Kulsoom, Saba Khan, Musab Saeed Siddiqui, Deedar Nabi

**Affiliations:** † Institute of Environmental Science and Engineering (IESE), School of Civil and Environmental Engineering (SCEE), National University of Sciences and Technology (NUST), H-12, 48000 Islamabad, Pakistan; ‡ GEOMAR Helmholtz Center for Ocean Research, Wischhofstrasse 1-3, 24148 Kiel, Germany

## Abstract

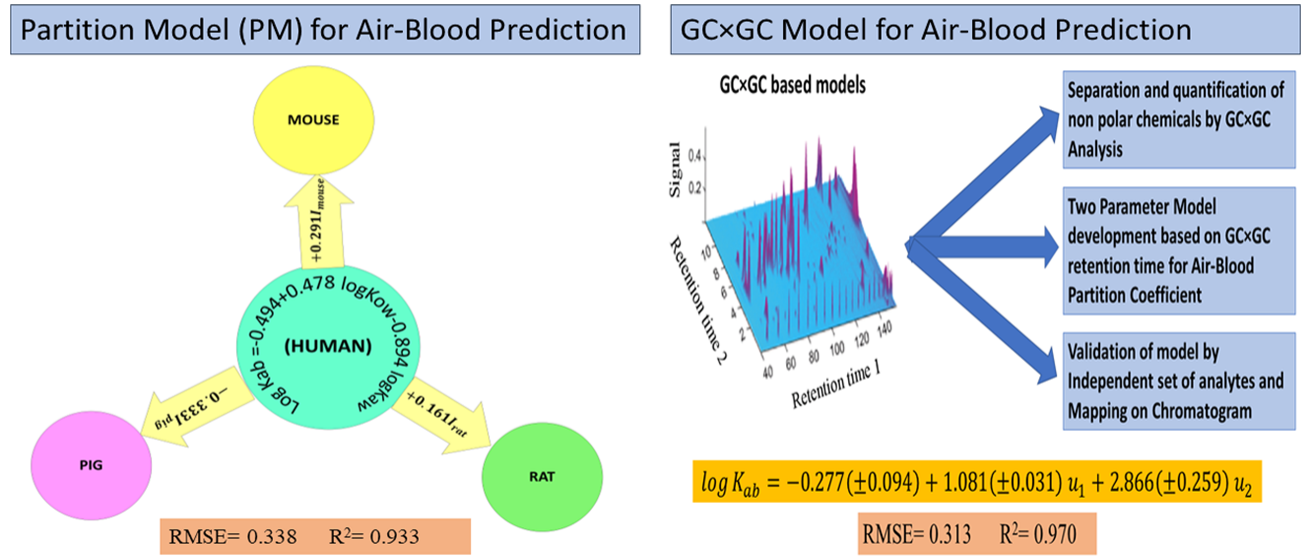

The air-blood partition coefficient (*K*_*ab*_) is extensively employed in human health risk assessment for chemical exposure. However, current *K*_*ab*_ estimation approaches either require an extensive number of parameters or lack precision. In this study, we present two novel and parsimonious models to accurately estimate *K*_*ab*_ values for individual neutral organic compounds, as well as their complex mixtures. The first model, termed the GC×GC model, was developed based on the retention times of nonpolar chemical analytes on comprehensive two-dimensional gas chromatography (GC×GC). This model is unique in its ability to estimate the *K*_*ab*_ values for complex mixtures of nonpolar organic chemicals. The GC×GC model successfully accounted for the *K*_*ab*_ variance (*R*^2^ = 0.97) and demonstrated strong prediction power (RMSE = 0.31 log unit) for an independent set of nonpolar chemical analytes. Overall, the GC×GC model can be used to estimate *K*_*ab*_ values for complex mixtures of neutral organic compounds. The second model, termed the partition model (PM), is based on two types of partition coefficients: octanol to water (*K*_*ow*_) and air to water (*K*_*aw*_). The PM was able to effectively account for the variability in *K*_*ab*_ data (*n* = 344), yielding an *R*^2^ value of 0.93 and root-mean-square error (RMSE) of 0.34 log unit. The predictive power and explanatory performance of the PM were found to be comparable to those of the parameter-intensive Abraham solvation models (ASMs). Additionally, the PM can be integrated into the software EPI Suite, which is widely used in chemical risk assessment for initial screening. The PM provides quick and reliable estimation of *K*_*ab*_ compared to ASMs, while the GC×GC model is uniquely suited for estimating *K*_*ab*_ values for complex mixtures of neutral organic compounds. In summary, our study introduces two novel and parsimonious models for the accurate estimation of *K*_*ab*_ values for both individual compounds and complex mixtures.

## 1 Introduction

The air-to-blood partition coefficient (*K*_*ab*_) is a biochemical parameter that governs the fate, behavior, and movement of volatile organic chemicals (VOCs) across various species in the animal kingdom. Specifically, *K*_*ab*_ plays a pivotal role in determining pulmonic gas exchange patterns. Alongside cardiac output and ventilatory flow, it regulates the inhalation of exogenous vapors (such as environmental contaminants or volatile anesthetic agents) and the elimination of endogenous chemicals through exhalation.^1^ Following inhalation, VOCs are conveyed to various organs within the body through the circulatory system. The transportation of these chemicals to different organs is contingent upon their capacity to partition from air to blood, as well as their ability to partition from blood to the respective body organs.^2^ Consequently, *K*_*ab*_ is widely used in chemical health risk assessment and the evaluation of the toxic potential of chemicals in humans and other animal species.^3^

Considering the importance and wide applications of *K*_*ab*_ it is mandatory to accurately determine its values. *K*_*ab*_ determination encompasses a range of methods including experimental techniques (in vivo and in vitro) and predictive models. In vivo methods^4−8^ directly measure a chemical’s concentration in the air and blood of living organisms. They provide highly accurate data and are commonly used in research and medical settings, offering advantages over in vitro or computational approaches.^9,10^ However, in vivo methods involving live animal testing are often restricted or regulated due to ethical concerns regarding animal welfare.^11^ Various regulatory bodies and authorities, such as World Organization for Animal Health (WOAH), REACH (Registration, Evaluation, Authorization, and Restriction of Chemicals), PETA (People for the Ethical Treatment of Animals), and Organization for Economic Co-operation and Development (OECD) impose limitations on these methods to promote the use of alternative approaches that minimize animal testing.^12,13^ Additionally, it is worth noting that approximately 90% of chemical drugs that undergo testing in animals fail to demonstrate efficacy during human clinical trials.^14,15^ Therefore, scientists and researchers are actively improving in vitro assays and advanced prediction models as ethical alternatives, providing reliable data while reducing the reliance on live animal testing.

In vitro methods for determining air to blood partition coefficients include artificial membrane models,^16^ liquid–liquid extraction,^17^ Parallel Artificial Membrane Permeability Assay (PAMPA),^18^ cell culture models,^19,20^ and Selected Ion Flow Tube Mass Spectrometry (SIFT-MS).^21,22^ Additionally, chromatographic techniques, like gas chromatography (GC) and liquid chromatography (LC),^23^ can also be used, with two-dimensional gas chromatography (GC×GC) being a powerful tool, offering enhanced resolution for volatile compound analysis and their complex mixtures.^24,25^ Despite avoiding live animal testing and reducing ethical concerns, in vitro testing has drawbacks like being costly, laborious, and complex.^26,27^

Hence, regulatory bodies recommend the development of prediction models as viable alternatives to avoid extensive experimentation.^28,29^ These models include Poly Parameter Linear Free Energy relationships (PP-LFERs)^2,30−32^ Quantitative Structure–Activity Relationship (QSAR) Models,^33,34^ Artificial Neural Network (ANN) Models,^35^ Random Forest (RF) Models,^36^ Genetic Algorithm (GA) Models,^35^ and hybrid approaches which are combination of two or more methods.^37^ However, each model has inherent limitations. PP-LFERs may necessitate extensive experimental data for parametrization and may lack accuracy in predicting complex chemical interactions. ANN models are prone to overfitting and may lack interpretability.^38^ RF Models exhibit constraints in handling nonlinear relationships and might be sensitive to data noise.^39^ GA Models, though effective, are computationally intensive and may converge to local optima, requiring parameter optimization.^35^ Recognizing these limitations is essential in selecting appropriate models and leveraging their strengths to enhance the accuracy of predictions for air-blood partition coefficients.

By considering all of these limitations of previous methods, in this research, we introduce two novel models that surpass the limitations of previous methods and deliver enhanced accuracy, applicability, and simplicity in predicting *K*_*ab*_ values.

The first type of our model for predicting *K*_*ab*_ values utilizes solute parameters *u*_1_ and *u*_2_ from a GC×GC chromatogram, based on our previous study.^40^ In the study conducted by Nabi et al.,^41^ favorable predictions of many environmental partitioning properties (log *K*) for nonpolar complex organic compounds were achieved using Linear Free Energy Relationships (LFERs) based on two solute parameters, *u*_1_ and *u*_2_. These parameters were extracted from the first- and second-dimension retention times of the analytes on a GC×GC chromatogram. The calibration of the GC×GC model (eq 1) was performed theoretically by using a set of 79 nonpolar model chemicals for 32 properties, followed by experimental validation by using a set of 52 nonpolar chemicals analyzed on the GC×GC instrument.
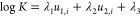
1Here λ_1_,λ_2_, and λ_3_ are partitioning system-specific parameters. The GC×GC model, demonstrated as a powerful tool in previous studies^24,26,27,40−43^ enables direct estimation of properties for detected nonpolar compounds in environmental mixtures. Nevertheless, to date, this approach remains unexplored for mapping *K*_*ab*_ values onto GC×GC chromatograms, presenting a significant opportunity for further investigation and advancement. Therefore, we created and tested a similar GC×GC model (eq 2) for the prediction of *K*_*ab*_ values.
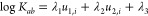
2

The GC×GC model we created holds significant potential as a powerful tool for accurately predicting *K*_*ab*_ values, particularly when applied directly to nonpolar compounds detected within environmental mixtures.

Our second model is Two Parameters Linear Free Energy Relationship (2P-LFER) which is based upon the air-to-water partition coefficients(*K*_*aw*_), and octanol-to-water partition coefficients(*K*_*ow*_), providing an alternative to the parameter-intensive Abraham Solvation Models (ASMs) which are referred to as PP-LFERs. ASMs are extensively developed and widely employed physicochemical descriptors-based models. These models have been utilized over the past few decades as reliable and robust gold standards for the prediction of various biochemical properties, including air-to-blood partition coefficients^2,32^ sensory irritation thresholds,^27,44−48^ blood-to-organs partition coefficients,^49−51^ skin permeability coefficients,^26,52^ and nonspecific lethal concentrations.^53^ Additionally, ASMs have demonstrated their efficacy as gold standards in predicting environmental properties such as standard enthalpies, solubilities, and interchangeable partition coefficients between air, water, soil, and organic matter.^54,55^*E, S, A, B, L,* and *V* are commonly referred to as Abraham Solute Descriptors (ASDs). These descriptors provide insights into intermolecular interactions. Specifically, (*E*) indicates polarizability, (*S*) represents a combination of polarizability and polarity, (*A*) signifies hydrogen bond acidity, (*B*) denotes hydrogen bond basicity, (*V*) characterizes the energy required for cavity formation during solvation, and (*L*) represents the gas to hexadecane partition coefficient, which is indicative of dispersion interactions.^56^ ASMs have previously been employed for the prediction of log *K*_*ab*_ values for a diverse range of chemicals in humans and select animal species.^2,32^ However, ASMs have a limitation in that they lack ASDs, with available descriptors currently limited to approximately 8000 chemicals. Although the ASD database is gradually expanding, the pace of growth is relatively slow compared to the vast number of registered industrial chemicals, which exceeds 65 million. Taking into account the aforementioned limitations of ASMs we developed the 2P-LFERs based on *K*_*aw*_ and *K*_*ow*_ (eq 3). Additionally, we aim to investigate any variations in log *K*_*ab*_ values between human and other mammalian species, such as rat, dog, mouse, rabbit, and pig, for which experimental log *K*_*ab*_ values are available.
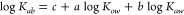
3

Here, *a*, *b*, and *c* are system constants resulting from regression analysis of log *K*_*ab*_, log *K*_*ow*_, and log *K*_*aw*_.

## 2 Methodology

### 2.1 Data Acquisition, Curation, and Transformation

The calibration and evaluation of the GC×GC model were conducted using data obtained from a prior study.^41^ The calibration set comprised 79 nonpolar chemicals, encompassing various families and functional groups of nonpolar organic compounds (Supporting Information, File 1, Table S1). The solute descriptors *u*_1_ and *u*_2_ for the 79 chemicals in the training set were derived by transforming the gas-stationary phase partition coefficients corresponding to the first- and second-dimension stationary phases of the GC×GC system. The values of the gas-stationary phase partition coefficients for these 79 chemicals were determined using the ASM equations published for the respective stationary phases.^27,57^ Subsequently, the calculated descriptors *u*_1_ and *u*_2_ were employed as independent variables, while log *K*_*ab*_ served as the dependent variable, to train the GC×GC model for predicting log *K*_*ab*_ values. In the subsequent step, a separate data set comprising 52 entirely independent nonpolar chemicals was utilized as a test set to validate the newly developed GC×GC model (Supporting Information, File 1, Table S2). For the independent test set, the retention times of nonpolar analytes in the first and second dimensions of the GC×GC system were converted into solute parameters, *u*_1_ and *u*_2_.^40,41^ Subsequently, the predicted log *K*_*ab*_ values for these 52 analytes were compared with both the available experimental values and the ASM predicted values to validate the GC×GC model. (Supporting Information, File 1, Table S2).

Due to the unavailability of experimental log *K*_*ab*_ values for a significant number of chemicals in both the GC×GC training set and validation set, estimated values from ASMs were utilized in those instances. This approach allowed for the development of the GC×GC model for log *K*_*ab*_ prediction in cases where experimental data were absent (Supporting Information, File 1, Table S2 and Table S3). Unlike ASMs, the GC×GC models have the advantage of being applicable to mixtures of nonpolar chemicals. Users can now estimate the log *K*_*ab*_ value of a chemical within a complex mixture of nonpolar organic compounds by simply providing the *u*_1_ and *u*_2_ values. These values can be easily measured using the GC×GC instrument and our MATLAB code.^58^ We have given a step-by-step guideline for the user’s facilitation to explain the complete procedure for the prediction of log *K*_*ab*_ values by using our GC×GC model (Supporting Information, File 2, Section S1). The GC×GC model training set and test set comprise a diverse range of nonpolar chemicals representing various chemical families. These families include *n*-alkanes, cycloalkanes, halogenated alkanes, benzene, linear alkylbenzenes, cycloalkenes, halogenated alkenes, halogenated benzenes, polycyclic aromatic hydrocarbons (PAHs), polybrominated diphenyl ethers (PBDEs), polychlorinated biphenyls (PCBs), polychlorinated naphthalenes (PCNs), and organochlorine pesticides. The inclusion of these different chemical families ensures a comprehensive representation of nonpolar compounds in the GC×GC model training and test sets.^27,41^

In our pursuit to develop *K*_*ow*_ and *K*_*aw*_ based 2P-LFERs, we compiled a data set of log *K*_*ab*_ values from the literature^2,31,32^ for various species: human (*n* = 157), rat (*n* = 134), dog (*n* = 15), pig (*n* = 5), mouse (*n* = 16), and rabbit (*n* = 17). This data set comprises 344 log *K*_*ab*_ values, which represent interspecies variations and include multiple entries for certain chemicals. The chemical classes covered in the data set are aliphatic hydrocarbons, cyclic hydrocarbons, simple and halogenated ethers, benzene derivatives, ethers, ketones, alcohols, and carbonic acid esters^2,31,32^ (Supporting Information, File 1, Table S4).

The Chemical Abstracts Service (CAS) numbers and Simplified Molecular Input Line Entry System (SMILES) codes for these chemicals were extracted from PubChem. Abraham solute descriptors (ASDs) were obtained from the UFZ LSER database,^59^ which is openly accessible. The log *K*_*aw*_ and log *K*_*ow*_ values, both experimental and estimated, were obtained using the HenryWin v3.20 and KOWWIN v1.69 modules of the EPI Suite v 4.11, an open-source software. Experimental log *K*_*ow*_ values were available for a total of 284 out of 344 data points, while the remaining 60 data points had their log *K*_*ow*_ values estimated using the ASM equations.^54,60^

In terms of log *K*_*aw*_, experimental values were available for 280 chemicals, and for the remaining 64 chemicals, predictions were made using ASM equations based on relevant literature.^54,61^

Our statistical analysis revealed that the ASM equations provided more accurate estimates for log *K*_*ow*_ (RMSE = 0.175) compared to KOWWIN v1.69 (RMSE = 0.291), as well as for log *K*_*aw*_ (RMSE = 0.162) compared to estimates from HenryWin v3.20. Therefore, we decided to use the ASM predicted values due to their superior accuracy, as determined by the analysis. For further details, see the Supporting Information, File 1, Table S5 and S6.

We recalibrated the Abraham solvation models (Supporting Information, File 2, Section S2) to enhance their predictive capabilities for log *K*_*ab*_ values. We observed a few discrepancies between certain values of Abraham Solvation Descriptors (ASDs) and their corresponding values reported in the most recent online database “LSER database for comptox users (2017)” available on the UFZ LSER database Web site. Consequently, we determined that the ASDs in question required upgrading to align with the latest reported values.^62^ Also, we found that the values of some ASDs taken from the literature^2,32^ were not even present in any of the previously published data sets available on the UFZ-LSER database (Supporting Information, File 1, Table S9). To rectify this issue, we employed the most recent version of the database, “LSER database for comptox users (2017)”, as a reference to correct these values (Supporting Information, File 1, Table S10).

## 3 Statistical Analysis

The statistical analyses including Multiple Linear Regression (MLR), Principal Component Analysis (PCA), Pearson correlation, and cross-validation tests were executed using RStudio (version 1.4.1106).^63^ To obtain the significant and optimal number of descriptors, we employed the stepwise MLR algorithm. This analysis was guided by various criteria, including the Variance Inflation Factor (VIF), Student’s *t* test, and Akaike Information Criteria (AIC). Additionally, we utilized Principal Component Analysis (PCA) to inspect the number of independent dimensions of chemical information in both the test and training data sets. The contribution of each variable in the principal components (PCs) was determined by conducting PCA on all of the variables. To explore the relationship between variables, we performed a Pearson correlation analysis. To assess the robustness of the models, we employed several cross-validation techniques including the bootstrap method (*n* = 1000), leave-one-out, K-fold cross-validation, Repeated K-fold cross-validation, and leave-one-out approaches. These techniques were utilized to ensure the reliability and generalizability of the models.

Furthermore, we conducted external validation by splitting the data set into a test set and a training set, maintaining a 20:80 ratio between the two subsets. This process allowed us to evaluate the performance of the models on independent data and assess their predictive capabilities. We used MATLAB Software^64^ to overlay the contours of log *K*_*ab*_ values onto GC×GC chromatograms by doing modification in a previous code.^27,58^

## 4 Results and Discussion

### 4.1 Comprehensive Two-Dimensional Gas Chromatography (GC×GC) based model for the prediction of log *K*_*ab*_ values

Our developed GC×GC model (eq 4) successfully captures the variations in log *K*_*ab*_ values for nonpolar compounds. The calibration data set consisted of 79 nonpolar chemicals. This data set comprised a combination of experimental values (*n* = 21) and log *K*_*ab*_ values predicted by Abraham Solvation Model (ASM) (*n* = 58).^32^ Additionally, for chemicals that had multiple experimental log *K*_*ab*_ values available, we calculated the average value to ensure consistency and reduce potential variability (Supporting Information, File 1, Table S 3).
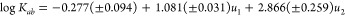
4*n* = 79, RMSE = 0.313, *R*^2^ = 0.970, Adj. *R*^2^ = 0.969, and *F* = 1215.

Analyzing the fitting coefficients and their associated standard errors enclosed in parentheses for both *u*_1_ and *u*_2_ in eq 4, it becomes evident that both the first and second dimensions of GC×GC play significant roles in elucidating the variability in air-blood partitioning for a representative set of nonpolar chemicals. Notably, the contribution of the second-dimensional stationary phase surpasses that of the first-dimensional phase by more than an order of magnitude in explaining the variation in log *K*_*ab*_ data. It may be deduced that a model based on the retention parameter of conventional one-dimensional gas chromatography would yield significantly lower accuracy when compared to the GC×GC model.

To establish the robustness and applicability of our GC×GC model (eq 4), we conducted both cross-validation and external validation tests. The results obtained from cross-validation, such as *R*^2^ values ranging from 0.962 to 0.980 and RMSE values ranging from 0.309 to 0.322, were highly consistent with the goodness-of-fit statistics of eq 4 (Supporting Information, File 1, Table S7). This indicates the robustness of our model and its ability to accurately predict log *K*_*ab*_ values.

To further validate our model, we utilized an independent data set consisting of 52 analytes representing diverse families of nonpolar organic compounds (Supporting Information, File 1, Table S2). Using eq 4, we estimated the log *K*_*ab*_ values for these analytes. The results were compared with the predictions obtained from the ASM. We found a strong agreement between the log *K*_*ab*_ values predicted by our GC×GC model and the ASM predictions, as evidenced by an RMSE value of 0.47 for the same data set of 52 nonpolar analytes. Overall, the cross-validation and external validation tests provide strong evidence of the reliability and accuracy of our GC×GC model in predicting log *K*_*ab*_ values for nonpolar organic compounds.

In our study, we employed the relationship described in eq 4 to map the log *K*_*ab*_ values by projecting the measured values of *u*_1_ and *u*_2_ onto a GC×GC chromatogram. This mapping was carried out for both the analyte test set, consisting of a mixture of nonpolar standards, and a mixture of short-chain chlorinated paraffins (SCCPs), which were previously analyzed.^41^ It is important to note that SCCPs are a complex mixture comprising approximately 7820 structurally distinct congeners.^65^

**Figure 1:**
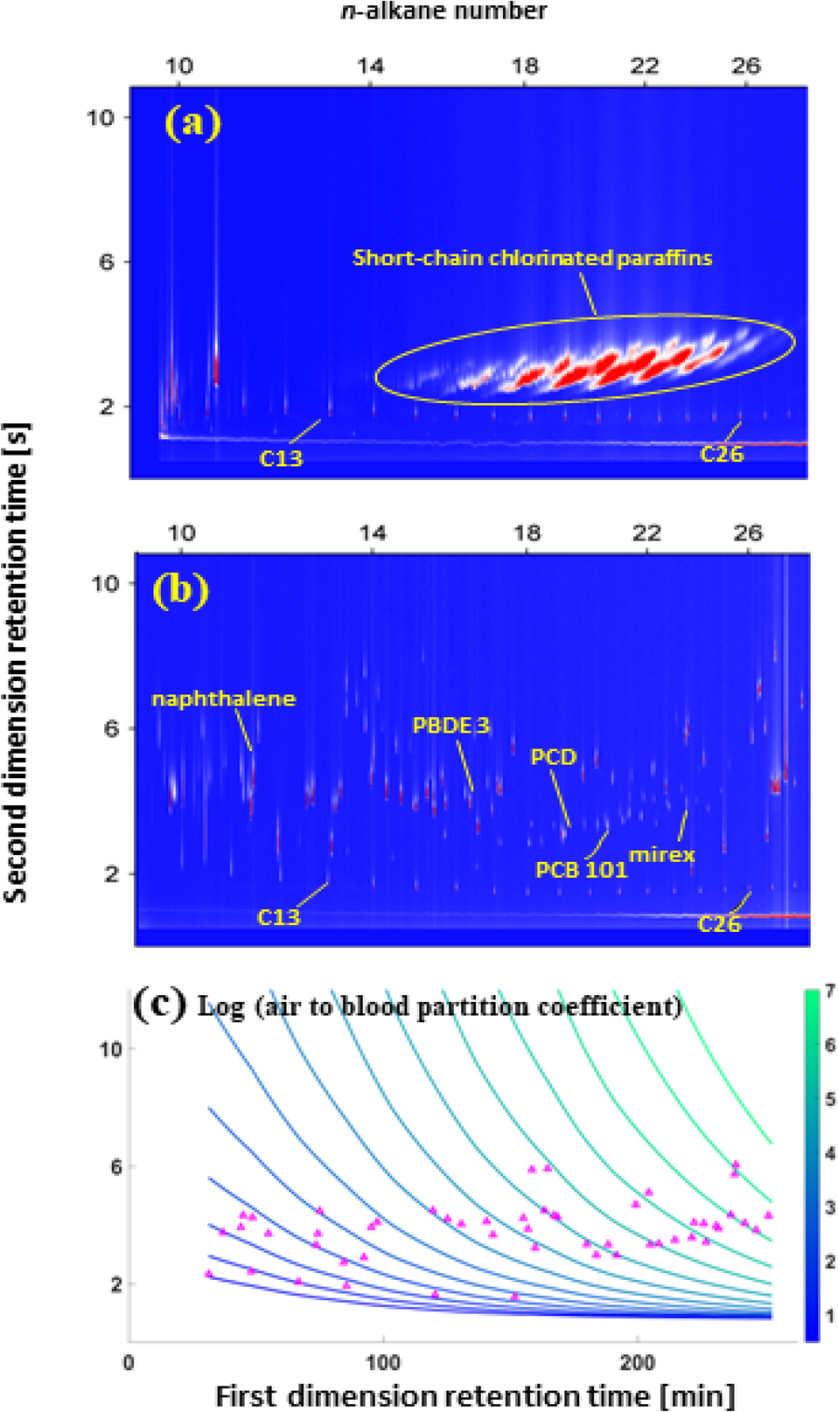
Mapping air to blood partition coefficients of nonpolar analytes onto a GC×GC chromatogram retention space. (a) represents the GC×GC chromatogram of a technical mixture of the short-chain chlorinated paraffins and (b) shows the GC×GC chromatogram of a mixture of injected standards, which were analyzed in a previous study.^41^ (c) Shows the contours of air to blood partitioning values of analytes in log units overlaid onto GC×GC chromatogram. In panel b, C26, C13, PBDE 3, PCB 101, and PCD respectively denote *n-*hexacosane, *n*-tridecane, 4-bromodiphenyl ether, 2,2′,4,5,5′-pentachlorobiphenyl, and 1,2,5,6,9-pentachlorodecane. (c) Pink triangles represent the 52 analytes of GC×GC model test set.

By projecting the measured *u*_1_ and *u*_2_ values onto the GC×GC chromatograms, we were able to assign log *K*_*ab*_ values to each discrete analyte detected within the nonpolar mixtures, such as the analyte test set used in our study (*n* = 52). This enabled us to identify and mark specific analytes as “chemicals of concern” based on their respective log *K*_*ab*_ values within the complex mixtures of nonpolar organic compounds (Figure 1b).

The GC×GC instrument possesses the capability of resolving complex mixtures into subgroups or ordered bands of congeners (Figure 1a). Our approach allows us to determine the relative trends of log *K*_*ab*_ values within these ordered bands, providing valuable insights into the partitioning behavior of congeners within the mixture.

It is worth noting that the concept of additive interactions of chemicals from air to blood has been supported by numerous previous studies,^66−69^ at least as an initial approximation, for estimating *K*_*ab*_ values of nonreactive chemical mixtures. Therefore, by utilizing the approach discussed in our study, it becomes possible to estimate the cumulative air-blood partitioning potential of a mixture of nonpolar organic chemicals.

Overall, our approach provides a valuable tool for evaluating the log *K*_*ab*_ values of individual analytes within complex mixtures, identifying chemicals of concern and estimating the cumulative air–blood partitioning potential of nonpolar organic chemical mixtures.

#### 4.1.1 Exploring Column Combinations for Enhanced Applicability of the GC×GC Model

The GC×GC column combination used in this study consisted of a nonpolar stationary phase in the first dimension, such as pure polydimethylsiloxane (e.g., DB-1), and a midpolar stationary phase in the second dimension, such as 50% diphenylsiloxane (e.g., Rtx-50). This column combination has been widely employed due to its effectiveness in achieving optimal structural separation of nonpolar organic chemical mixtures, including compounds such as polychlorinated alkanes (PCAs), polychlorinated biphenyls (PCBs), hydrocarbons, and polybrominated diphenyl ethers (PBDEs).^27,70,71^ This column combination captures the important intermolecular interaction descriptors—i.e., *S*, *E*, *L*, *V*—needed to describe the 99% partitioning variation of nonpolar chemicals.^57,71^

To investigate if there are commercially available stationary phases that exhibit larger magnitudes of system coefficients (*s*, *e*, *l*, and *v*) and are therefore more suitable and accurate in sensing *S*, *E*, *L*, and *V* properties of nonpolar compounds, we conducted a thought experiment. We considered different options for the second dimension column by examining the Abraham solvation system constants available for extensively used commercial stationary phases, such as poly(ethylene glycol) (e.g., HP-INNOwax), bis(cyanopropylsiloxane)-*co*-methylsilarylene (e.g., HP-88), and 50% diphenylsiloxane (e.g., Rtx-50).^72^ Our analysis revealed that the magnitudes of the *l* and *e* coefficients for stationary phases such as HP-88 and HP-INNOwax were comparable to those of Rtx-50, while the magnitudes of the *s* and *a* coefficients for those phases were higher than those reported for the Rtx-50 column.

To further assess the impact of different column combinations on the estimation of log *K*_*ab*_ values, we trained the GC×GC model equation using the calculated retention factors of a calibration set of chemicals (*n* = 79) on six column combinations, including DB-1×HP-88, DB-1×HP-INNOwax, DB-1×Rtx-50, HP-5×HP-INNOwax, HP-5×Rtx-50, and HP-5×HP-88. The results of our analysis demonstrated that there were no significant changes in the statistics when switching the column combination, apart from the one used in our study (Supporting Information, File 1, Table S8). Furthermore, it is worth noting that bis(cyanopropylsiloxane)-*co*-methylsilarylene and poly(ethylene glycol) stationary phases exhibit thermal instability beyond 250 and 260 °C, respectively. As a result, poly(methylphenylsiloxane), which has a maximum temperature limit of 350 °C, is preferred for eluting high boilers and is a suitable alternative to these phases.

In order to broaden the applicability domain of the GC×GC model, which is currently limited to nonpolar chemicals, we explored the inclusion of polar analytes by considering alternative column combinations. We examined a total of 103 stationary columns ranging from conventional phases to ionic liquid phases. The determination of retention factors for the chemicals in the ASM training data set of log *K*_*ab*_ was performed using the system coefficients described in literature.^57,73^

After evaluating the performance of various column combinations, we identified (1-propyl-1-methylpiperidinium bis(trifluoromethylsulfonyl) imide and 1,9-di(3-[2-hydroxyethyl]limidazolium) nonane bis(trifluoromethylsulfony)imide) as the most suitable column combination for accurately predicting log *K*_*ab*_ values for both polar and nonpolar analytes.

These column combinations, incorporating ionic liquid phases, demonstrate the potential to expand the GC×GC model’s applicability to include a broader range of analytes encompassing both polar and nonpolar compounds.

### 4.2 Formulation of Partition Models

#### 4.2.1 Theoretical Rationalization of Partition Model

Our hypothesis revolves around the idea that the two-dimensional partition coefficients (PM), which are based on log *K*_*ow*_ and log *K*_*aw*_, bear a strong resemblance to the five-dimensional ASM in their capacity to capture the variations in log *K*_*ab*_ values across a diverse set of organic compounds. To put this hypothesis to the test, our initial approach involved an exploration of the information content embedded in the six ASDs, namely, *E*, *S*, *A*, *B*, *L*, and *V*—within the ASM framework—and how this aligns with the information content encoded in the PM descriptors log *K*_*ow*_ and log *K*_*aw*_, particularly concerning their association with log *K*_*ab*_.

**Figure 2:**
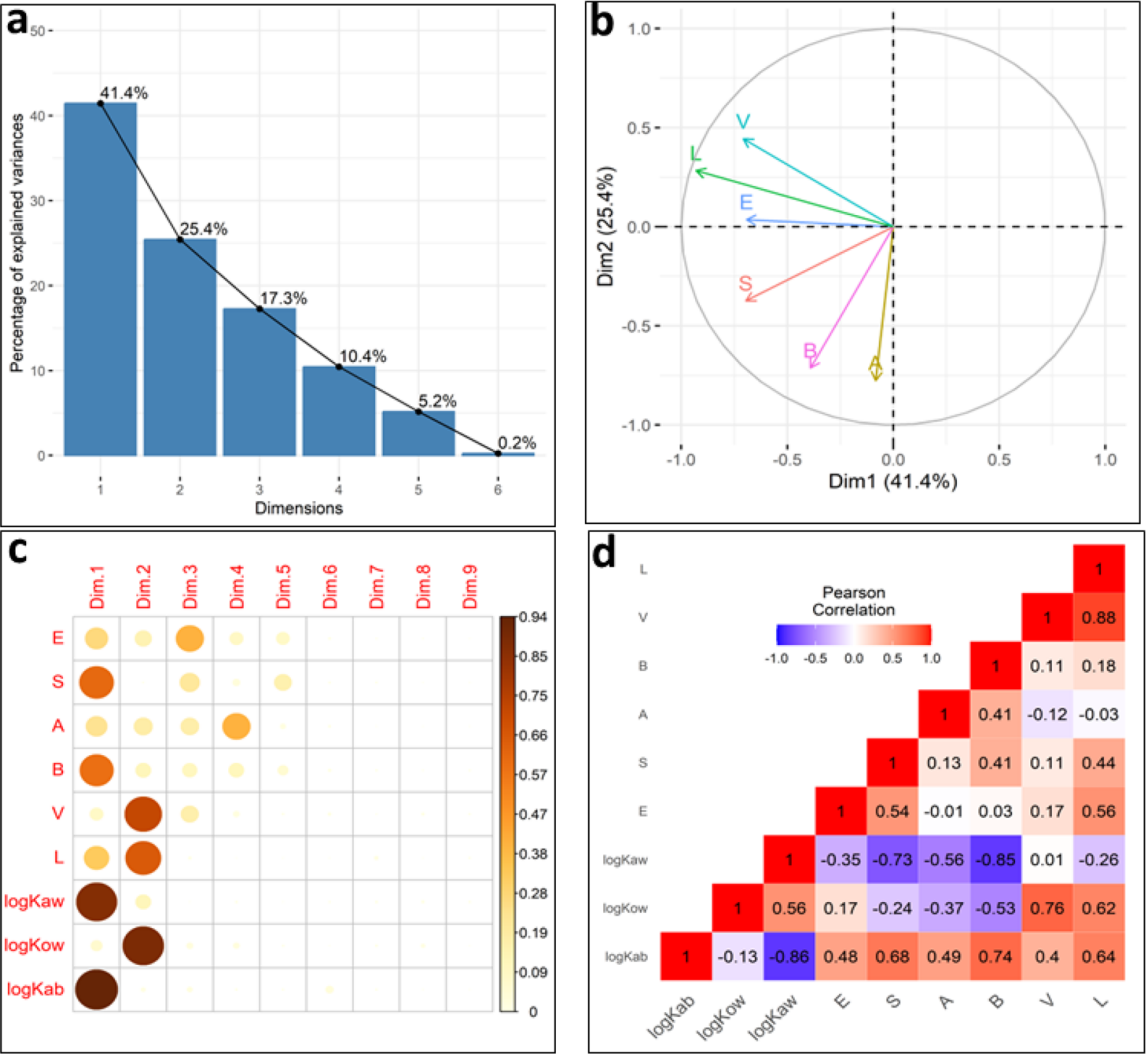
represents the dimensionality analysis for the Partition Model (PM) training set. The top panels illustrate the results of Principal Component Analysis (PCA) performed on a 344 × 6 matrix, [*E*, *S*, *A*, *B*, *L*, *V*]. (a) Scree plot of eigenvalues is shown, which indicates the amount of variation captured by each principal component. (b) The correlation circle visually represents the quality of representation and the relationships among the variables in the first two dimensions. The angles between the arrow lines indicate the correlation between the ASDs. The length of the arrows corresponds to the quality of the representation of each descriptor. Panel c displays the distribution of the quality of representation across nine dimensions obtained through PCA of a 344 × 9 matrix, [*E*, *S*, *A*, *B*, *L*, *V*, log *K*_*ab*_, log *K*_*ow*_, and log *K*_*aw*_]. The sizes of the circles and the intensity of the colors are proportional to the quality of representation of each parameter. (d) Panel represents the correlation matrix obtained from a Pearson correlation analysis performed on a 344 × 9 matrix, [*E*, *S*, *A*, *B*, *L*, *V*, log *K*_*ab*_, log *K*_*ow*_, and log *K*_*aw*_]. The values of *r* (correlation coefficient) indicate the magnitude of the correlation between the variables.

For the above, we conducted PCA on a 344 × 6 matrix ([*E*, *S*, *A*, *B*, *L*, *V*]) comprising the ASDs of the calibration set of log *K*_*ab*_. The results from PCA revealed that out of the six dimensions considered here, the first two dimensions corresponded to 66.8% of the total information (as illustrated in Figure. 2a). Specifically, we discovered that the first dimension in the PCA analysis was primarily influenced by the linear combination of *E*, *V*, and *L*, accounting for approximately 40.4% of the information. Additionally, the contributions of the *A* and *B* descriptors were relatively minor in this dimension. On the other hand, the second dimension was predominantly shaped by the *S*, *A*, and *B* descriptors, with smaller contributions from other ASDs, encompassing approximately 25.4% of the information (Figure. 2b).

Next, we included log *K*_*aw*_, log *K*_*ow*_, and log *K*_*ab*_ in the above PCA test to investigate their correspondence to the ASDs. This resulted in a 344 × 9 matrix, [*E*, *S*, *A*, *B*, *L*, *V*, log *K*_*aw*_, log *K*_*ow*_, log *K*_*ab*_]. PCA indicated that among these nine dimensions, the first two dimensions explained 77% of the data set. The three partition coefficients, log *K*_*ow*_, log *K*_*aw*_, and log *K*_*ab*_, were primarily associated with the first two dimensions (Figure. 2c). Furthermore, a Pearson correlation analysis of all descriptors (*E*, *S*, *A*, *B*, *L*, *V*, log *K*_*aw*_, log *K*_*ow*_, and log *K*_*ab*_) indicated that log *K*_*aw*_ and log *K*_*ow*_ exhibited moderate to strong correlations with ASDs and log *K*_*ab*_ (Figure 2d).

Based on these findings, we concluded that log *K*_*aw*_ and log *K*_*ow*_ are suitable descriptors that can serve as potential alternatives to ASDs. They can effectively explain the variability in log *K*_*ab*_ data and accurately predict the log *K*_*ab*_ values of VOCs within the scope of this study.

Beyond the statistical evidence presented above, the following empirical considerations also influenced the selection of log *K*_*aw*_ and log *K*_*ow*_ as key parameters in this study. Blood is a complex mixture composed of diverse macromolecules, including lipids, proteins, carbohydrates, and nucleic acids.^74,75^ Octanol has been frequently employed as a surrogate for these macromolecules, primarily because of the moderate to strong correlation between log *K*_*ow*_ and the partition coefficients of these macromolecules.^76,77^ Although the literature has proposed various one-parameter linear free energy relationships, their accuracy across diverse chemical families frequently does not match the performance of ASMs.^78,79^ This suggests that a single parameter such as log *K*_*ow*_ may be inadequate to capture the partitioning variability for these macromolecules. The rationale for adopting log *K*_*ow*_ in the PM stems from the similarity between the octanol–water partitioning system and the cellular composition of blood. Similarly, the selection of log *K*_*aw*_ is influenced by its relevancy to the blood plasma composition, which is approximately 50% water. This resemblance facilitates a more accurate estimation of the partitioning behavior of VOCs, mirroring the transition of VOCs from air to water and, consequently, from air to blood.

#### 4.2.2 Formulation and Validation of Partition Model for Prediction of log *K*_*ab*_

The PM is based on the linear combination of log *K*_*aw*_ and log *K*_*ow*_ explained variation in the log *K*_*ab*_ data successfully (*n* = 344, *R*^2^ = 0.927, Adjusted *R*^2^ = 0.927, RMSE = 0.351, *F* = 2177). Additionally, we employed indicator variables to formulate a model equation (eq 5) that encompasses species other than humans.
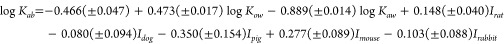
5*n* = 344, RMSE = 0.338, *R*^2^ = 0.934, Adj. *R*^2^ = 0.932, and *F*= 947.

The indicator variables for *I*_*dog*_ and *I*_*rabbit*_ were found to have insignificant coefficients with *P* > 0.05 and large standard errors. As a result, these indicator variables were excluded from the formulation of the eq (eq 6).
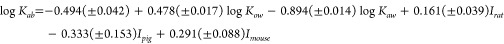
6*n* = 344, RMSE = 0.338, *R*^2^ = 0.933, Adj. *R*^2^ = 0.932, and *F* = 946.

Equation 6 was formulated without including any insignificant indicator variables, making it preferable over eq 5. To assess the internal robustness of our model (eq 6), we conducted four types of cross-validation tests: the leave-one-out approach, bootstrap method, K-fold cross-validation, and repeated K-fold cross-validation. The results of these tests are shown in Supporting Information, File 1, Table S11, indicate strong internal validation statistics (*R*^2^ = 0.930–0.936 and RMSE = 0.337–0.342), which closely match the regression statistics of eq 6.

For external validation, we randomly divided the data into a training set (*n* = 275) and a test set (*n* = 69), maintaining a proportion of 80:20. From the training set, we derived eq 7 using 275 chemicals.
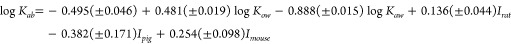
7*N* = 275, RMSE = 0.337, *R*^2^ = 0.933, Adj. *R*^2^ = 0.932, and *F* = 748

*n*_*external*_ = 69, *R*^2^_*external*_ = 0.946, and RMSE_*external*_ = 0.361.

The regression statistics and fitting coefficients of both eq 6 and eq 7 demonstrate statistical similarity, indicating that our partition model is robust against variations in the data. Furthermore, the calculated values of RMSE*_external_* and *R*^2^*_external_*, obtained from comparing the predicted log *K*_*ab*_ values for the test set (*n*_*external*_ = 69) using eq 7 with the corresponding experimental values, provide evidence that the partition model is reliable and robust for predicting interspecies (human, rat, mouse, and pig) log *K*_*ab*_ values for additional neutral organic chemicals.

In our final analysis, we examined the influence of filling missing values with ASM-predicted log *K*_*ow*_ and log *K*_*aw*_ values on the performance of the PM. The PM trained on the extended data set (*n* = 344) (eq 5), which included both experimental and ASM-estimated values for log *K*_*ow*_ and log *K*_*aw*_ showed slightly improved performance (*R*^2^ = 0.934, RMSE = 0.338, *F*-statistic = 946.93). In contrast, the model trained solely on experimental data (*n* = 239) for log *K*_*ow*_ and log *K*_*aw*_ yielded an *R*^2^ = 0.911, RMSE = 0.364, and *F*-statistic = 479.42. However, the differences in regression coefficients and their associated standard errors between these two cases of fittings are not significantly different, as illustrated in Figures S1 and Tables S1 and S2. Given that the extended data set (*n* = 344) provides a more extensive range for Abraham solute descriptors, log *K*_*ow*_, log *K*_*aw*_, and log *K*_*ab*_ as depicted in Figure S2, the PM derived from this larger data set (eq 5) is recommended.

According to eq 6, the log *K*_*ab*_ values for mice and rats were found to be statistically higher than those for humans by 0.29 and 0.16 log units, respectively. Similarly, the negative offsets observed for the pig data set indicate that the log *K*_*ab*_ values for pig are lower than those for humans by 0.33 log units. Consequently, our analysis reveals that larger animals demonstrate a lower tendency for chemical absorption into the bloodstream compared to smaller animals. This observation could be attributed to the faster respiration or breathing rate in smaller species, as the diffusion of volatile organic compounds (VOCs) occurs from areas of high concentration to areas of low concentration. Thus, an increase in pulmonary blood flow rate and respiratory rate enhances systemic absorption.^74^

We successfully demonstrated and validated the suitability and practicality of our proposed partition model for predicting log *K*_*ab*_ values across various species. The statistical analysis further confirms that our model can yield results comparable to those obtained from parameter-intensive ASMs (Supporting Information, File 1, Table S12). This supports the reliability and effectiveness of our approach in predicting log *K*_*ab*_ values for different species.

#### 4.2.3 Scientific and Theoretical Premise of Partition Models

The transfer of a solute from air to blood phase is directed by various intermolecular forces such as Debye dipole-induced dipole forces (represented by *E* descriptor), London dispersion forces (represented partially by the *V, L,* and *E* descriptors), Keesom dipole–dipole forces (encoded in the *S* descriptor), and hydrogen bonding forces (represented by *A* and *B* descriptors).^80^ The dimensionality analyses, including Pearson correlation and principal component analyses, provide valuable insights into the role of different factors in the partitioning process. Based on these analyses, it is evident that log *K*_*ow*_ effectively captures certain interactions such as Debye dipole-induced dipole forces, London dispersion forces, and cavitation energy of solvation. These interactions are relevant for understanding the transfer of solutes between water and macromolecules such as proteins and lipid phases of the blood.

However, log *K*_*ow*_ alone does not sufficiently account for Keesom dipole–dipole forces and hydrogen bonding interactions. This is supported by the moderate correlations observed between log *K*_*ow*_ and the descriptors *S* (*r* = −0.24), A (*r* = −0.37), and *B* (*r* = −0.53), which are associated with these specific intermolecular interactions.

On the other hand, the air–water partition system exhibits a strong responsiveness to Keesom dipole–dipole forces and hydrogen bonding interactions. This is evident from the significant correlations observed between log *K*_*aw*_ and the descriptors *S* (*r* = −0.73), *A* (*r* = −0.56), and *B* (*r* = −0.85), as shown in Figure 2d. These intermolecular interactions play a crucial role in describing the polar interactions of chemicals transferring from the air phase to the blood phase, as is evident by the strong correlations between log *K*_*ab*_ and ASDs (Figure 2d).

Overall, these findings highlight that an understanding of the partitioning process can be achieved by considering a linear combination of both air–water and octanol–water partition coefficients, as they collectively account for the intermolecular interactions involved in the transfer of chemicals between air and blood.

#### 4.2.4 Feasibility of Integrating PM into EPI Suite

Estimation Programs Interface Suite (EPI Suite) is a Windows-based program containing multiple modules used for the prediction of physical/chemical properties of several compounds and their fate estimation. It is a widely used screening tool, especially for chemicals that lack experimental data.

In our study, we explored the feasibility of integrating a new module into the EPI Suite for predicting log *K*_*ab*_ values across different species. To achieve this, we utilized the predicted log *K*_*aw*_ and log *K*_*ow*_ values obtained from EPI Suite modules, namely, Henrywin v3.21 and KOWWIN v1.69, and incorporated them into the partition model (PM). We then compared the log *K*_*ab*_ values estimated by PM using this approach with the corresponding experimental values reported in the literature.^2^ The statistical assessment revealed satisfactory results with a root-mean-square error (RMSE) of 0.57. These findings support the integration of the partition model into EPI Suite, as it enables users to obtain reasonably accurate estimations of log *K*_*ab*_ values through the air-blood partition coefficients estimation module.

We assessed the performance of EPI Suite integrated PM for chemicals with strong hydrogen bonding interactions. Within the PM training data sets, we compared experimental values against predictions from EPI Suite and ASM for a subset comprising chemical families like ketones, alcohols, esters, and ethers. For log *K*_*ow*_ values, both EPI Suite and ASM predictions closely matched experimental data (*n* = 52), with RMSEs of 0.17 and 0.16 log units, respectively. However, the EPI Suite predictions of log *K*_*aw*_ exhibited an RMSE of 0.46 log units when compared to 56 available experimental observations. The ASM predictions for the same data set resulted in an RMSE of 0.23 log units. This higher estimation error of the EPI Suite predicted log *K*_*aw*_ may lead to inflated residuals (predicted versus observed) of log *K*_*ab*_ for these chemical families. Table S12 highlights such cases where PM exhibits greater residuals than ASM, for chemicals known for strong hydrogen bonding. Users should be aware of this limitation when using PM with EPI Suite data.

In summary, our study demonstrates the successful integration of the partition model into the EPI Suite, allowing users to reliably estimate log *K*_*ab*_ values using the air-blood partition coefficients estimation module.

## 5 Domain of Applicability for GC×GC Model and PM

The predictions of the PM, and GC×GC models are more reliable for simple and small molecules than for complex chemicals with multiple functional groups and/or high hydrophobicity values (log *K*_*ow*_ > 6), extremely low volatility, or very low aqueous solubility. These complex chemicals may exhibit significant uncertainties in the input data, such as log *K*_*ow*_ and log *K*_*aw*_ for PM, and *u*_1_ and *u*_2_ for GC×GC model. Nevertheless, most of the predicted values by PM were within 1 log unit of the experimental values, except for a few deviations. In the case of PM, decane, nonane, 2,3-dimethylbutane, 2-methoxyethanol, tetrafluoromethane, and dodecane showed deviations from the anticipated regular trend (Supporting Information, File 1, Table S13). The uncertainty could be attributed to the experimentally measured values of log *K*_*ab*_, as the experimental values of nonane and 2,3-dimethylbutane significantly differed from their back-calculated values obtained from the ASM and PM equations. The experimental value of nonane also diverged from the experimental log *K*_*ab*_ values of other linear alkanes. Similarly, the experimental value of 2,3-dimethylbutane (0.780) did not agree with the back-calculated values and differed significantly from the experimental value of 2,2-dimethylbutane (−0.590), which was in good agreement with the back-calculated values from the model equations. Although these two chemicals differ by only one carbon atom in the methyl group, the difference in their experimental values suggests a possible error in the experimental value of nonane. Additionally, decane and dodecane exhibited much higher experimental log *K*_*ow*_ values (6.10 and 5.01, respectively), indicating their hydrophobic nature. For the other outliers, it is possible that their published descriptors (log *K*_*ow*_ and log *K*_*aw*_) may involve significant errors, considering that their experimental log *K*_*ab*_ values align closely with those of other chemicals within the same functional groups. However, the predicted values of log *K*_*ab*_ by PM deviate from this trend.

The GC×GC model, utilizing the present column combination of polydimethylsiloxane (e.g., DB-1) in the first dimension and 50% diphenylsiloxane (e.g., Rtx-50) in the second dimension, is suitable only for nonpolar analytes. Furthermore, chemicals that exhibit significant localized hydrogen bonding interactions may demonstrate larger deviations from the experimental values. The GC×GC model predictions for both the calibration set and a completely independent test set of analytes align well with the experimental and ASM calculated values, except for heptachlor (Supporting Information, File 1, Table S2, and Table S3).

## 6 Outlook and Limitations

The GC×GC column combination utilized in this study, consisting of pure poly(dimethylsiloxane) (e.g., DB-1) in the first dimension and 50% diphenylsiloxane (e.g., Rtx-50) in the second dimension, was specifically calibrated for nonpolar chemicals. However, in Section 4.1.1, we have proposed alternative column combinations that can be employed for polar chemicals, enabling the development of GC×GC models for the estimation of log *K*_*ab*_ values in such cases. Furthermore, due to limited data availability for individual species, a single model was created by averaging all of the accessible experimental log *K*_*ab*_ values for each chemical. Given the availability of experimental data for each species, it would be possible to develop separate GC×GC models for all species in the future, utilizing the same approach described in this study.

The PM developed in this study and the published ASM are designed to predict the behavior of only neutral compounds only. These models do not apply to ionic species as the air-to-blood partitioning mechanisms for charged species differ significantly. However, it is worth considering the inclusion of descriptors such as p*K*_*a*_ (acid ionization constant) in future models, as this could expand their applicability to include ionic species.^27^

Furthermore, the use of ASDs in previous correlation equations for ASMs limits the estimation of log *K*_*ab*_ values to a restricted number of compounds, as experimental ASD values are available for only around 8000 chemicals. In order to account for interspecies variability in air-to-blood partitioning correlations, we incorporated indicator variables into the PMs (eq 5 and eq 6). However, due to limited available experimental data for log *K*_*ab*_ values, separate model equations could not be developed for most species. Only human and rat data sets provided a sufficient amount of data to develop separate robust and reliable model equations. Therefore, we opted to incorporate available data for different species into a single model equation using the indicator variables.

In conclusion, the parsimonious PM offer accurate predictions of log *K*_*ab*_ values comparable to the more parameter-intensive ASMs by employing the commonly available partition coefficients, namely *K*_*aw*_ and *K*_*ow*_. These two parameters not only serve as descriptors in the PM equations but also provide theoretical and mechanistic insights by capturing the necessary intermolecular interactions, as detailed in Section 4.2.3. One advantage of PMs is their capability to predict log *K*_*ab*_ values for chemicals where ASDs are unavailable. The use of indicator variables in PMs facilitates the examination of interspecies variability in air-to-blood data and enables the comparison of predicted values among different species. Another notable feature of PMs is their compatibility with integration into the EPISuite software as a separate module, ensuring reliable estimation of log *K*_*ab*_ values.

The GC×GC model is distinctive in its ability to predict log *K*_*ab*_ values for complex mixtures of neutral nonpolar chemicals. To the best of our knowledge, no previous approach based on physicochemical parameters existed for predicting the log *K*_*ab*_ values of chemical mixtures. Therefore, our models address certain limitations of previous approaches and present two innovative methodologies for predicting the air-to-blood partitioning of individual chemicals and their complex mixtures.

## Data Availability

Data, including all molecular structures and their properties, is available in a machine-readable format as Supporting Information. The GC×GC MATLAB code is available on request from the authors.

## Supporting Information Available

File S1: Tables of a calibration set of nonpolar chemicals, validation and training sets for GC×GC model development, experimental and predicted partition coefficients, cross-validation tests, column combination analysis results, unmatched and updated descriptor values, and comparisons of ASMs and PMs predicted log *K*_*ab*_ values against experimental values(XLSX)
File S2: Guidance on predicting log *K*_*ab*_ values through the GC×GC model and insights from the analysis and enhancement of ASMs for log *K*_*ab*_ prediction (PDF)
