# Supplementary material for: Novel Models for Accurate Estimation of Air–Blood Partitioning: Applications to Individual Compounds and Complex Mixtures of Neutral Organic Compounds
Source: J Chem Inf Model. 2023 Nov 13;63(22):7056–66. doi: 10.1021/acs.jcim.3c01288 (PMC10685450; doi:10.1021/acs.jcim.3c01288)
Supplement: Supplementary file 2 — ci3c01288_si_002.pdf [file ci3c01288_si_002.pdf]

Supporting Information, File 2

Novel Models for Accurate Estimation of Air-Blood Partitioning: Applications to Individual Compounds and Complex Mixtures of Neutral Organic Compounds

Ahmad Aakash[1], Ramsha Kulsoom[1], Saba Khan[1], Musab Saeed Siddiqui[1], Deedar Nabi[1, 2*]

[1]Institute of Environmental Science and Engineering (IESE), School of Civil and Environmental Engineering (SCEE), National University of Sciences and Technology (NUST), H-12, 48000 Islamabad, Pakistan.

[2] GEOMAR Helmholtz Center for Ocean Research, Wischhofstrasse 1-3, 24148 Kiel, Germany

*Corresponding author email: dnabi@geomar.de

Contents

Section S1: How to predict $logKab$ values of nonpolar analytes using GC×GC model 1

Section S2: Analysis and upgradation of ASMs for prediction of $logKab$ values of different species 2

Section S3: Reevaluation of Partition Model with Experimental Octanol-Water and Air-Water Data 6

References 8

Section S1: How to predict $logK_{ab}$ values of nonpolar analytes using GC×GC model.

Readers can follow the steps given below to estimate the $\log K_{ab}$values of nonpolar analytes detected on the GC×GC chromatogram using the MATLAB code, which can be assessed from the authors.

1. Obtain optimum GC×GC separation for your sample of interest using the same or equivalent column combination considered in this study.

2. Identify at least15 nonpolar calibration analytes, in addition to an n-alkane series, within the elution window of interest of the GC×GC chromatogram. These analytes may already be present or added in the sample or may be analyzed separately as standard using the same GC×GC instrumental program.

3. Provide the first- and second-dimension retention times, and ASDs of calibration analytes as an input to the MATLAB code.

4. Query the MATLAB code to predict the $\log K_{ab}$ values based on the first- and second-dimension retention times of nonpolar analytes detected on the GC×GC chromatogram. Users may also directly overlay the contours of these $\log K_{ab}$ values onto GC×GC chromatogram.

Section S2: Analysis and upgradation of ASMs for prediction of $logK_{ab}$ values of different species

We recalibrated the Abraham solvation models to enhance their predictive capabilities for $log K_{ab}$ values. We observed discrepancies between certain values of Abraham Solvation Descriptors (ASDs) and their corresponding values reported in the most recent online database "LSER database for comptox users (2017)" available on the UFZ LSER database website. Consequently, we determined that the ASDs in question required upgrading to align with the latest reported values [1]. Also, we found that the values of some ASDs taken from the literature [2], [3] were not even present in any of the previously published datasets available on the UFZ-LSER database (Supporting Information, Table S 7).We identified discrepancies in the recorded values of certain parameters, suggesting errors in their acquisition or reporting. To rectify this issue, we employed the most recent version of the database, "LSER database for comptox users (2017)," as a reference to correct these values. (Supporting Information, Table S 8).

Following the dataset update comprising 358 chemicals, we proceeded to redefine the equations of ASMs using the ESABL model and Goss modified Abraham equation SABVL. To account for $logK_{ab}$ values of species other than humans, we introduced indicator variables. Notably, the $logK_{ab}$ dataset for humans served as the reference, rendering the utilization of indicator variables unnecessary for human $logK_{ab}$ values (Equations 1-4).

$$logK_{ab} = -1.175(\pm 0.041) + 0.449(\pm 0.070)\mathrm{E} + 1.107(\pm 0.082)\mathrm{S} + 3.677(\pm 0.167)\mathrm{A} + 2.789(\pm 0.094)\mathrm{B} + 0.372(\pm 0.015)\mathrm{L} + 0.160(\pm 0.034)I_{rat} - 0.058(\pm 0.080)I_{dog} - 0.339(\pm 0.136)I_{pig} + 0.303(\pm 0.076)I_{mouse} - 0.087(\pm 0.075)I_{rabbit} + 0.231(\pm 0.146)I_{hamster} - 0.485(\pm 0.171)I_{horse} - 0.097(\pm 0.205)I_{guineapig} - 0.281(\pm 0.149)I_{sheep} + 0.028(\pm 0.290)I_{cat} \quad (1)$$

N= 358 RMSE= 0.286 R^2= 0.953 Adj. R^2 = 0.951 F= 465

The variables N, RMSE, R^2, Adj. R^2, and F represent the total number of data points, root mean square error, and coefficient of determination, adjusted R^2, and the F-test value of model significance, respectively. Indicator variables were employed to incorporate the $logK_{ab}$ values of different species into a unified model equation. The indicator variables were denoted by (I) with subscripts representing the corresponding species names. For example, I_{rat} was utilized to include the $logK_{ab}$ values of rats in Eq. 1. These variables are arbitrary and allow users to activate or deactivate specific species, enabling the estimation of their $logK_{ab}$ values using a single equation. The indicator variables were assigned values of 1 or 0. To calculate the $logK_{ab}$ values for horses using Eq. 1, we set I_{horse}=1 and all other indicator variables to 0. The goodness-of-fit statistics for Eq. 1 showed an improvement of 0.004 in R^2 and a decrease of 0.007 log units in RMSE compared to the previous version of this model published in the literature [2]. The improvement in the correlation can be attributed to the correction of certain ASD values, which were replaced with the most up-to-date values obtained from the UFZ LSER database website. The standard errors of each coefficient are presented in parentheses, indicating that the coefficients corresponding to the indicator variables used for $logK_{ab}$ datasets of dog, rabbit, hamster, guinea pig, sheep, and cat were found to be statistically insignificant. Consequently, the indicator variables for these species were eliminated, and a new regression analysis was performed. As a result, a new ASM equation (Eq. 2) was formulated, incorporating four indicator variables.

$$logK_{ab} = -1.190(\pm 0.036) + 0.469(\pm 0.068)\mathrm{E} + 1.086(\pm 0.081)\mathrm{S} + 3.668(\pm 0.166)\mathrm{A} + 2.811(\pm 0.092)\mathrm{B} + 0.373(\pm 0.014)\mathrm{L} + 0.172(\pm 0.033)I_{rat} - 0.312(\pm 0.135)I_{pig}$$

$$+0.314(\pm 0.075)I_{mouse} - 0.459(\pm 0.171)I_{horse} \quad (2)$$

N= 358 RMSE= 0.287 R^2= 0.952 Adj. R^2 = 0.951 F= 769

The goodness of fit statistics almost remained the same by removing the statistically insignificant indicator variable coefficients. The coefficients of the indicator variables indicate the extent of difference in $logK_{ab}$ values between humans and other mammals such as rats, pigs, mice, and horses. These coefficients suggest that there is a statistically significant difference in $logK_{ab}$values between humans and other mammals. Although this difference is relatively small, it should be carefully assessed considering the importance and sensitivity of human health risk assessment. Furthermore, we conducted a re-evaluation of the performance of the Goss modified ASM (SABVL) using 358 data points. The objective was to determine the most suitable variant of ASM for predicting $logK_{ab}$values.

$$logK_{ab} = -0.722(\pm 0.070) + 0.790(\pm 0.094)S + 3.403(\pm 0.164)A + 2.841(\pm 0.089)B - 1.381(\pm 0.154)V + 0.718(\pm 0.036)L + 0.148(\pm 0.033)I_{rat} - 0.024(\pm 0.076)I_{dog} - 0.243(\pm 0.130)I_{pig} + 0.256(\pm 0.130)I_{mouse} - 0.059(\pm 0.071)I_{rabbit} + 0.186(\pm 0.139)I_{hamster} - 0.360(\pm 0.164)I_{horse} - 0.115(\pm 0.195)I_{guineapig} - 0.208(\pm 0.142)I_{sheep} + 0.012(\pm 0.276)I_{cat} \quad (3)$$

N= 358 RMSE= 0.273 R^2= 0.958 Adj. R^2 = 0.956 F= 514

Equation 3 exhibits superior statistical performance in comparison to Equation 1. However, similar to Equation 1, some indicator variables' coefficients are found to be statistically insignificant. Therefore, we conducted another multiple linear regression (MLR) analysis, this time considering only the significant indicator variables, ASDs, and $logK_{ab}$values. The result of this analysis is presented as Equation 4.

$$logK_{ab} = -0.713(\pm 0.066) + 0.760(\pm 0.091)S + 3.385(\pm 0.161)A + 2.860(\pm 0.087)B + 0.731(\pm 0.035)L - 1.433(\pm 0.150)V + 0.155(\pm 0.032)I_{rat} - 0.220(\pm 0.129)I_{pig} + 0.260(\pm 0.072)I_{mouse} - 0.337(\pm 0.163)I_{horse} \quad (4)$$

N= 358 RMSE= 0.273 R^2= 0.957 Adj. R^2 = 0.956 F= 859

Equation 4 shows slight differences in terms of the goodness of fit statistics compared to previous equations, indicating an improved predictive power. Notably, there is an increase in the R^2 value and a decrease in the RMSE value, indicating better accuracy in predicting $logK_{ab}$

values. Based on these improvements, we recommend users to prioritize Equation 4 among the available ASMs for predicting $logK_{ab}$values of both humans and the other species included in this equation. It is important to note that although the offsets between human and rat (0.16 log units), human and pig (0.22 log units), human and mouse (0.26 log units), and human and horse (0.34 log units) in the $logK_{ab}$data are relatively small, they should not be ignored. These differences, although minor, could have potential implications and should be taken into consideration during data analysis and risk assessment related to human health.

Section S3: Reevaluation of Partition Model with Experimental Octanol-Water and Air-Water Data

Initially, we combined both experimental and predicted values of the partition coefficients of octanol-water and air-water to create an extended dataset for training the air-blood partition model, aiming to enhance its applicability, especially in cases where experimental data are sparse. To inspect the influence of this combination on the model, we conducted an additional analysis using solely the experimental values. The statistics obtained from this focused analysis are detailed below.

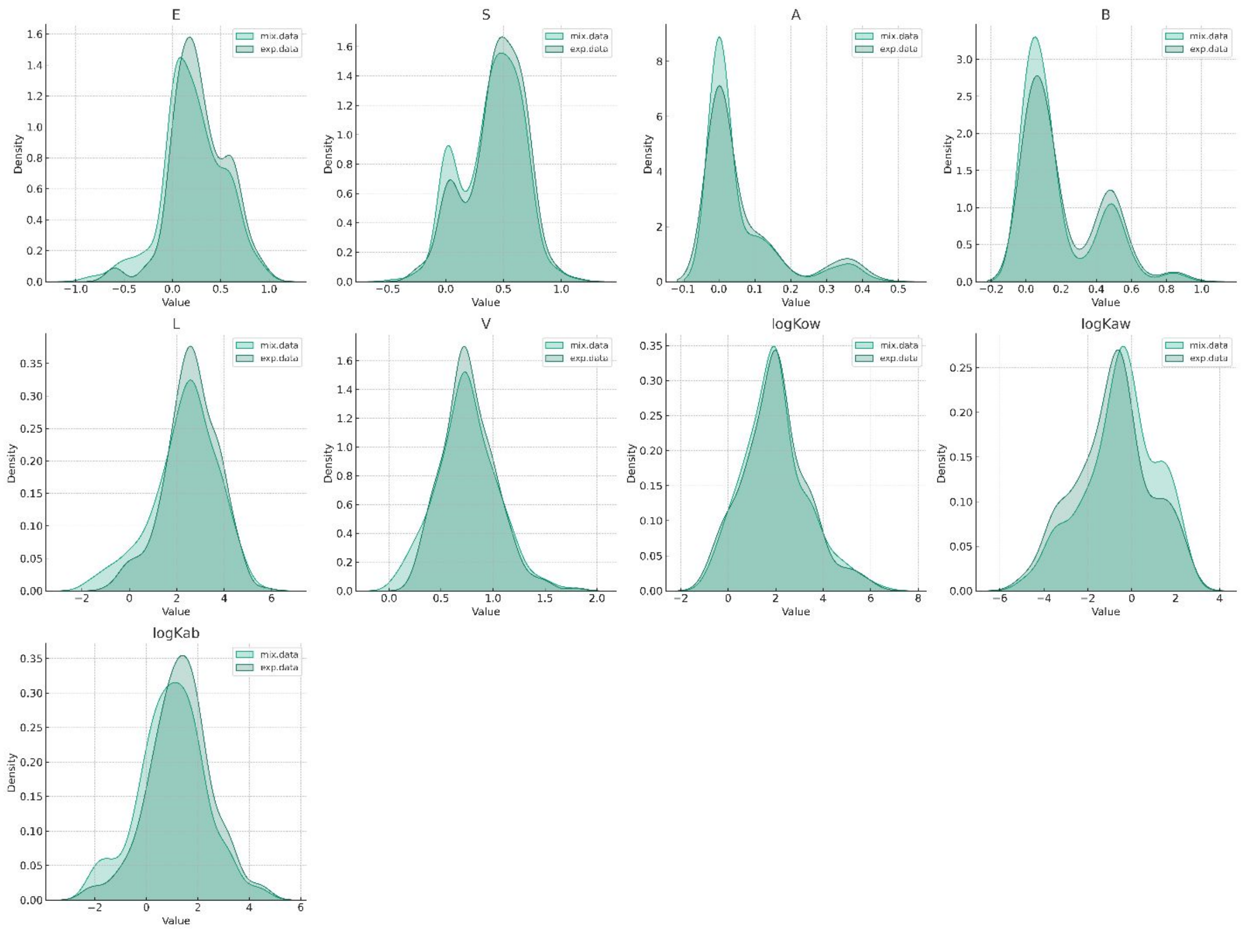

Figure S1: Kernel Density Estimation plots comparing the distribution of Abraham solute descriptors (E, S, A, B, V, and L) and partition coefficients for the octanol-water, air-water, and air-blood systems across two datasets: mix.data (blue) and exp.data (orange). The mix.data set includes both experimental values and those of logKow and logKaw estimated using the Abraham Solvation Model to fill missing entries. In contrast, exp.data comprises only experimental logKow and logKaw values.

Table S1: Regression Summary for the PM based on experimental and estimated values of logKow and logKaw

Predictor	Coefficient	Std. Error	t-value	p-value	95% Conf. Int. Low	95% Conf. Int. High
const	-0.494	0.042	-11.790	4.E-27	-0.576	-0.411
**logKow**	0.478	0.017	28.615	3.E-92	0.445	0.511
**logKaw**	-0.894	0.014	-65.683	2.E-194	-0.921	-0.867
**Irat**	0.161	0.039	4.077	6.E-05	0.083	0.238
**Ipig**	-0.333	0.153	-2.170	3.E-02	-0.635	-0.031
**Imouse**	0.291	0.088	3.296	1.E-03	0.117	0.464

R-squared	Adjusted R-squared	RMSE	N	F-statistic
**0.933**	0.932	0.338	344	946.93

Table S1: Regression Summary for the PM based on experimental data of logKow and logKaw

Predictor	Coefficient	Std. Error	t-value	p-value	95% Conf. Int. Low	95% Conf. Int. High
const	-0.375	0.068	-5.541	8.E-08	-0.508	-0.242
**logKow**	0.437	0.025	17.790	3.E-45	0.388	0.485
**logKaw**	-0.874	0.020	-44.544	6.E-116	-0.913	-0.836
**Irat**	0.128	0.049	2.591	1.E-02	0.031	0.225
**Ipig**	-0.394	0.260	-1.515	1.E-01	-0.905	0.118
**Imouse**	0.304	0.110	2.765	6.E-03	0.088	0.521

R-squared	Adjusted R-squared	RMSE	N	F-statistic
**0.911**	0.909	0.3640	239	479.42

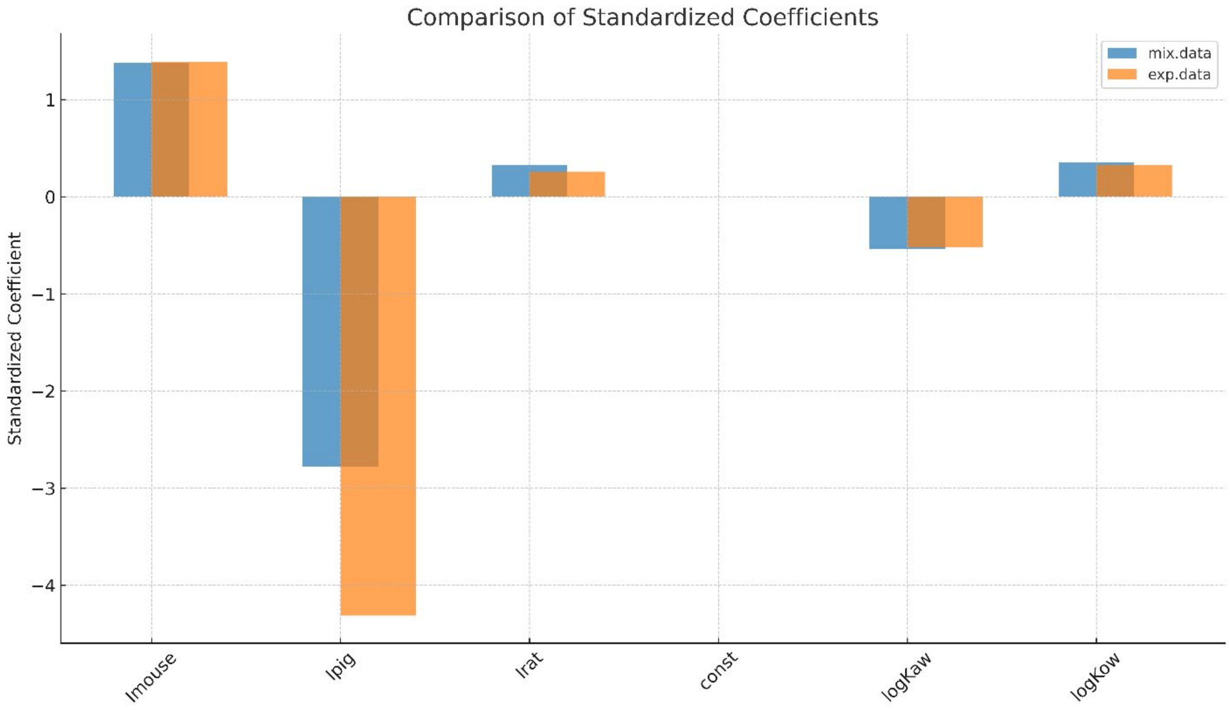

Figure S2: Comparison of standardized coefficients for the PM. The blue bars represent the PM trained on mix data (combining experimental and estimated values of logKow and logKaw), while the orange bars depict the PM trained exclusively on experimental data (exp.data) for logKow and logKaw.

References

(1) Endo S, Watanabe N, Ulrich N, Bronner G, G. K.-U. *http:// www. ufz. de/ lserd.Accessed 03 October 2022*.

(2) Sprunger, L. M.; Gibbs, J.; Acree, W. E.; Abraham, M. H. Correlation of Human and Animal Air-to-Blood Partition Coefficients with a Single Linear Free Energy Relationship Model. *QSAR Comb. Sci.* **2008**, *27*, 1130–1139. https://doi.org/10.1002/qsar.200860078.

(3) Abraham, M. H.; Ibrahim, A.; Acree, W. E. Air to Blood Distribution of Volatile Organic Compounds: A Linear Free Energy Analysis. *Chem. Res. Toxicol.* **2005**, *18*, 904–911. https://doi.org/10.1021/tx050066d.
